# New Surfactant with SP-B and C Analogs Gives Survival Benefit after Inactivation in Preterm Lambs

**DOI:** 10.1371/journal.pone.0047631

**Published:** 2012-10-16

**Authors:** Matthias Seehase, Jennifer J. P. Collins, Elke Kuypers, Reint K. Jellema, Daan R. M. G. Ophelders, Olga L. Ospina, J. Perez-Gil, Federico Bianco, Raffaella Garzia, Roberta Razzetti, Boris W. Kramer

**Affiliations:** 1 Department of Pediatrics, Maastricht University Medical Center, Maastricht, The Netherlands; 2 Research and Development Department, Chiesi Farmaceutici SpA, Parma, Italy; 3 Department of Biochemistry, Faculty of Biology, Complutense University, Madrid, Spain; 4 Department of Physics, Pontificia Universidad Javeriana, Bogota, Colombia; The Ohio State Unversity, United States of America

## Abstract

**Background:**

Respiratory distress syndrome in preterm babies is caused by a pulmonary surfactant deficiency, but also by its inactivation due to various conditions, including plasma protein leakage. Surfactant replacement therapy is well established, but clinical observations and *in vitro* experiments suggested that its efficacy may be impaired by inactivation. A new synthetic surfactant (CHF 5633), containing synthetic surfactant protein B and C analogs, has shown comparable effects on oxygenation in ventilated preterm rabbits versus Poractant alfa, but superior resistance against inactivation *in vitro*. We hypothesized that CHF 5633 is also resistant to inactivation by serum albumin *in vivo*.

**Methodology/Principal Findings:**

Nineteen preterm lambs of 127 days gestational age (term = 150 days) received CHF 5633 or Poractant alfa and were ventilated for 48 hours. Ninety minutes after birth, the animals received albumin with CHF 5633 or Poractant alfa. Animals received additional surfactant if P_a_O_2_ dropped below 100 mmHg. A pressure volume curve was done post mortem and markers of pulmonary inflammation, surfactant content and biophysiology, and lung histology were assessed. CHF 5633 treatment resulted in improved arterial pH, oxygenation and ventilation efficiency index. The survival rate was significantly higher after CHF 5633 treatment (5/7) than after Poractant alfa (1/8) after 48 hours of ventilation. Biophysical examination of the surfactant recovered from bronchoalveolar lavages revealed that films formed by CHF 5633-treated animals reached low surface tensions in a wider range of compression rates than films from Poractant alfa-treated animals.

**Conclusions:**

For the first time a synthetic surfactant containing both surfactant protein B and C analogs showed significant benefit over animal derived surfactant in an *in vivo* model of surfactant inactivation in premature lambs.

## Introduction

Respiratory distress syndrome (RDS) is a significant cause of morbidity and mortality in preterm infants [1]–[4]. RDS is caused by a deficiency, dysfunction, or inactivation of pulmonary surfactant [5], [6]. Surfactant lowers surface tension and improves pulmonary dynamic compliance. Numerous surfactants of either animal extract or synthetic design have been developed and tested [7]. Although both synthetic and animal derived surfactant preparations have been shown to be beneficial, studies comparing animal derived surfactant preparations to synthetic preparations have demonstrated improvement in immediate ventilator support, decreased risk of pneumothorax, and decreased risk of mortality in infants receiving the animal derived products [7]. Furthermore, there is a marginal decrease in chronic lung disease among preterm newborns treated with animal derived surfactant preparations when compared to the synthetic preparations [7]. Therefore, surfactants from animal derivation including porcine lung extracts such as Poractant alfa (Chiesi Farmaceutici SpA, Parma, Italy) are currently the most often used ones in preterm infants [8].

However the respiratory failure in preterm infants is not only due to a primary surfactant deficiency but is also caused by surfactant inactivation as a result of plasma proteins leaking into the airways from areas of epithelial disruption and injury [9]. Various conditions which often affect preterm infants, such as exposure to chorioamnionitis, pneumonia, sepsis, meconium aspiration and asphyxia, may lead to surfactant inactivation [9]. Surfactant inactivation of preparations such as Poractant alfa has already been studied in several animal models of RDS. Calkovska and colleagues reported the physiological parameters of ventilated preterm rabbits after surfactant inactivation and treatment with Poractant alfa [10].

Mechanisms of inactivation include impairment of surfactant interfacial adsorption due to steric barriers imposed by serum and/or inflammatory proteins [11], [12] and impairment of compressibility properties of surfactant films due to incorporation of spurious components like cholesterol, lysophospholipids or bile salts [13].

Both surfactant proteins SP-B and SP-C have been proposed to participate in optimizing the surface behavior of surfactant under the demanding conditions imposed by the respiratory physiology [14] and in particular both SP-B [15], [16] and SP-C [17], [18] have been reported to increase the resistance of surfactant to inactivation by various agents. CHF 5633 is a fully synthetic surfactant containing two phospholipids and two peptides analogues of human surfactant proteins B and C, designed to be resistant to oxidative injury. The phospholipids in CHF 5633 consist of a mixture of DPPC, the most important phospholipid in terms of physiologic function and POPG sodium salt (POPG, Na) which has been reported to inhibit lung inflammation [19], [20]. Furthermore, Sato et al. demonstrated a superior oxygenation and lung compliance in ventilated preterm lambs treated with CHF 5633 compared to other, animal-derived surfactant preparations [21]. Based on these features we hypothesized that CHF 5633 could better counterbalance surfactant inactivation upon replacement and would improve oxygenation and lung function in preterm babies with RDS.

## Methods

### Animals

The study was approved by the Animal Ethics Research Committee, Maastricht University, The Netherlands (animal ethics protocol 2010-129). Texel ewes were date-mated and the fetuses were randomized to receive Poractant alfa or CHF 5633 before inactivation with albumin. Due to ethical reasons we did not include control animals, which were treated with CHF5633 or Poractant alfa only without surfactant inactivation, as previous studies already demonstrated the physiological parameters of ventilated preterm lambs after Poractant alfa or CHF5633 treatment [21], [22]. Experiments were conducted in 19 preterm lambs of both genders at a gestational age of 127 days (term = 150 days).

### Surfactant preparations

CHF 5633 is a synthetic surfactant comprising dipalmitoyl-phosphatidylcholine (DPPC), 1-palmitoyl-2-oleoyl-sn-glycero-3-phosphoglycerol (POPG), sodium salt, synthetic surfactant protein C analog (IPSSPVHLKRLKLLLLLLLLILLLILGALLLGL) and synthetic surfactant protein B analog (CWLCRALIKRIQALIPKGGRLLPQLVCRLVLRCS) as active ingredients. The final product is a sterile suspension with a total concentration of 80 mg/ml. As control treatment, the animal derived surfactant Poractant alfa (Curosurf®, 80 mg/ml, Chiesi Farmaceutici SpA, Parma, Italy) was used, which is frequently used in clinical practice. All preparations were supplied by Chiesi Farmaceutici SpA (Parma, Italy).

### Experimental protocol

The pregnant ewes underwent cesarean section under epidural and local subcutaneous analgesia with 2% lidocaine. In addition, they were sedated with 1 mg midazolam intravenously which was repeated if necessary. After a lower midline incision, the fetus was carefully extracted through a small incision of the uterus. An endotracheal tube (4.5 mm) was inserted and catheters were placed in the umbilical artery and in the jugular vein and used for baseline blood sampling (Abbott i-STAT 1 Blood Gas Analyzer, Abbott Laboratories, Illinois, USA) and for continuous monitoring of fetal mean arterial blood pressure (MABP) and heart rate (HR).

After the umbilical cord was cut, the fetus was brought to an open, heated incubator (IW930 Series CosyCot™ Infant Warmer, Fisher & Paykel Healthcare, Auckland, New Zealand) maintaining a body temperature of 38°C. The lambs were connected to intermittent positive pressure ventilation (IPPV) using a ventilator Babylog 8000 (Dräger, Lübeck, Germany) with initial settings as follows: FiO_2_ = 1, PEEP 8 cmH_2_O, PIP 25 cmH_2_O, frequency 60/min, I∶E 1∶2. Thereafter, inspiratory pressure was increased by 2 cmH_2_O if P_a_CO_2_ was higher than 80 mmHg or by 4 cmH_2_O if P_a_CO_2_ was higher than 110 mmHg. When a maximum PIP of 40 cmH_2_O was reached, no further adjustments were made. These ventilation settings, which are rather aggressive compared to clinical ventilation settings, were chosen to test the performance of the surfactant preparations under unfavorable conditions and to minimize the variations in ventilation settings between the individual animals. The lambs were ventilated for 48 hours. Sedation was maintained with ketamine (4 mg/kg/h) and midazolam (50 µg/kg/h). For nutrition a solution of 20% glucose and Ringer's lactate was used in a concentration allowing for an infusion rate of 1–3 mL/kg/h. After 2 hours of life a urinary catheter was placed to monitor urine production and kidney function.

### Surfactant inactivation protocol

**Figure 1:**
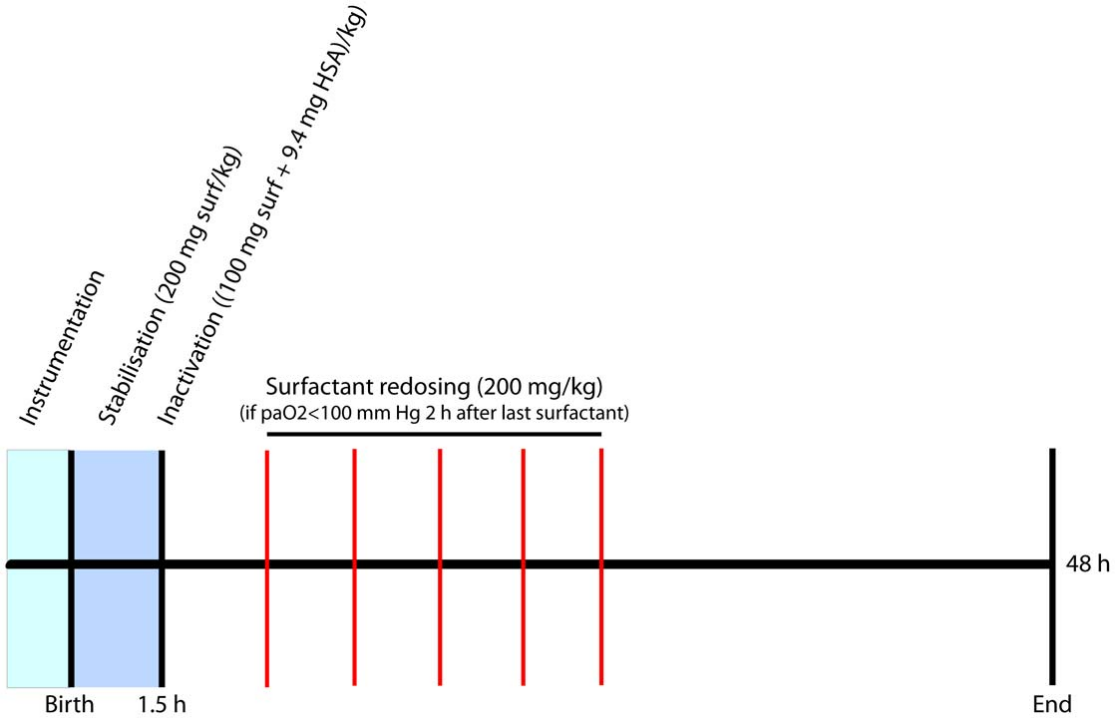
Study design. Preterm lambs received a 200 mg/kg bodyweight intra-tracheal dose of either Poractant alfa or CHF 5633. Ninety minutes after birth a mixture consisting of 9.4 mg/kg human serum albumin and either 100 mg/kg Poractant alfa or 100 mg/kg CHF 5633 was given intra-tracheally to simulate surfactant inactivation. If P_a_O_2_ dropped below 100 mmHg, the lambs received an additional dose of either 200 mg/kg Poractant alfa or 200 mg/kg CHF 5633 every two hours until the P_a_O_2_ increased above the 100 mmHg threshold. 48 hours after birth the lambs were euthanized.

Before being connected to IPPV, preterm lambs received either CHF 5633 or Poractant alfa intra-tracheally in a dosage of 200 mg/kg BW (Figure 1). Executors of the experiments were completely blinded for the type of surfactant given: vials were wrapped in aluminium foil and marked as “A” or “B” by DRMGO (blinding was only lifted after all experiments and analyses had been performed). Ninety minutes after birth a mixture consisting of 9.4 mg/kg human serum albumin (optimal dose was determined in prior dose-effect experiments) and either 100 mg/kg CHF 5633 or 100 mg/kg Poractant alpha was given intra-tracheally. The surfactant was used to provide an even spread of the inactivator in the preterm lung. Blood gases were taken every hour. If P_a_O_2_ dropped below 100 mmHg, the lambs received every two hours an additional dose of either 200 mg/kg CHF 5633or 200 mg/kg Poractant alfa until the P_a_O_2_ was again higher than 100 mmHg.

### Humane endpoints

The lambs were euthanized by an i.v. injection of 2 ml T61® (Veterinaria AG, Zürich, Switzerland) after 48 hours or earlier if reaching a human endpoint. Humane endpoints were defined as cardiovascular failure (arterial pH<6.6, heart rate<100 beats per minute and/or mean arterial blood pressure<30 mmHg) despite maximum dobutamine administration (20 µg/kg/min), or if either of these situations occurred in the presence of edema combined with no response to furosemide treatment (1 mg/kg) and kidney failure.

### Post-mortem measurements

The thorax was opened to perform a post-mortem pressure volume curve. The endotracheal tube, which was used for ventilation during life, was connected to a manometer and glass syringe. The lungs were inflated until a maximum pressure of 40 cm H_2_O was reached and the corresponding gas volume was recorded. Subsequently, the pressure was reduced to 20, 15, 10, 5 and 0 cm H_2_O and corresponding gas volumes were recorded. The lungs were removed from the chest and weighed. The left lung was lavaged three times with 20 mL 0.9% NaCl. The volume of the resulting bronchoalveolar lavage fluid (BALF) was recorded and aliquots were used for differential cell counts or snap frozen for surfactant analysis. The right upper lobe (RUL) was inflation-fixed at 30 cmH_2_O in 10% formalin for 2 hours for histological analysis.

### H&E staining

Hematoxylin and eosin staining was performed on paraffin embedded RUL lung sections (4 µm, transverse). The sections were deparaffinized in an ethanol series and incubated in Mayer's hematoxylin for 5 minutes. After rinsing in running water for 10 minutes, the sections were incubated in an eosin solution for 1 minute. Subsequently the sections were dehydrated in an ethanol series and coverslipped. Evaluation was performed by light microscopy (Leica DM2000) with LeicaQWin Pro v.3.7.0 software (Leica Microsystems, Wetzlar, Germany).

### Disaturated phospholipid analysis in BALF

The disaturated phospholipids content was measured in BALF of the left lung. Briefly, 1.2 mL of BALF was thawed and centrifuged for 10 minutes at 300 times g-force/relative centrifugal force (rcf). One mL of the resultant liquid phase was transferred to a clean glass tube and evaporated overnight at 60°C. The remaining BALF condensate was dissolved in 1 mL of carbontetrachloride and osmiumtetraoxide (1∶10) solution, aided by the addition of a glass bead and intermittent vortexing, and evaporated for 1.5 hours at 60°C. The condensate was dissolved in 1 mL chloroform∶methanol (20∶1) and transferred to a column filled with glass wool impregnated with 0.8 g activated aluminium oxide. The columns were subsequently flushed with 8 mL chloroform∶methanol (20∶1), followed by 5 mL chloroform∶methanol∶ammonia (35∶15∶1). After passage through the column, the liquids were collected and evaporated at 60°C until only BALF condensate remained. After dissolving the condensate in 1 mL chloroform, 500 µL FeSCN was added. After shortly vortexing to ensure a homogenous mixture, the samples were centrifuged for 10 minutes at 600 g. The 150 µl of the resulting chloroform phase was transferred to a 96 well plate and scanned at 488 nm using a Multiskan Spectrum spectrophotometer (Thermo Fisher Scientific, Waltham, USA) and Scanit RE for MSS 2.2 software. The disaturated phospholipid concentration could then be calculated by using a standard dilution series.

### Hemoglobin spectrometry

To quantify the hemorrhagic aspect of BALF, 200 µl of each sample was scanned at 410 nm wavelength using a Multiskan Spectrum spectrophotometer and Scanit RE for MSS 2.2 software. The resulting optical density (OD) was then corrected for bodyweight.

### Ventilation efficiency index (VEI) and P/F ratio

The ventilation efficiency index (VEI) was calculated as VEI = 3,800/(respiratory rate×[PI_max_−PEEP]×P_a_CO_2_], where 3,800 is a CO_2_ production constant ([ml×mmHg]/[kg×min]) [23]. As a measure for oxygenation, ratios were calculated by dividing the PaO_2_ by FiO_2_ (FiO_2_ = 1).

### Biophysical analysis by captive bubble surfactometry

To analyze the functional behaviour of surfactant samples from CHF 5633 and Poractant alfa treated animals, material obtained from the bronchoalveolar lavage was tested in a custom-built captive bubble surfactometer (CBS), as described elsewhere [13], [24]. Lavages were first centrifuged at 40000 g to obtain the large aggregates of pulmonary surfactant complexes and phospholipids were quantitated from pellets by phosphorus analysis [25]. Samples were then diluted to 25 mg/mL phospholipid concentration and 200 nL were injected onto the surface of an air bubble of 50 µL formed in the chamber of the CBS, using a subphase Tris 5 mM pH 7 containing NaCl 150 mM and 10% sucrose, thermostated at 37°C and subjected to continuous stirring. Continuous monitoring of bubble shape with a video camera allowed determination of surface tension. Once the sample adsorbed to equilibrium surface tension (initial adsorption, IA), the bubble was expanded to a volume of 150 µL to allow for surfactant re-adsorption (post-expansion adsorption, PEA) during 5 min. Then, the bubble was subjected to quasi-static compression-expansion cycling, in which the bubble size was first reduced and then enlarged in a stepwise fashion during four consecutive quasi-static cycles. Finally, after 1 min delay, compression-expansion dynamic cycling started, in which the bubble size was continuously varied at 20 cycles/min, which is a speed comparable with breathing rates. Illustrative surface tension-relative area isotherms are presented after repeating at least three experiments with each of the samples, and averaged relevant parameters such as minimal surface tension at the end of compression, the percent of area reduction required to reach minimal tension, and the maximal tension upon expansion, were compared for each group.

### Data Analysis

Results are given as means±standard error of mean (SEM). The groups were compared using a Mann-Whitney u-test or a two-way repeated-measures analysis of variance where appropriate. Survival analysis was performed with a Gehan-Breslow-Wilcoxon test. Statistical analysis was performed by GraphPad Prism v5.0. Significance was accepted at p<0.05.

## Results

At birth fetal lambs from the two different treatment groups were similar in birth weight and umbilical artery pH (Table 1). The female to male ratio was also similar at 5∶2 for the CHF 5633 group compared to 5∶3 in the Poractant alfa group. 4 preterm lambs were not deemed physically healthy and were therefore not included into the experiment shortly after birth.

**Table 1:** Observations of preterm lambs born at 127 days GA which were treated with Poractant alfa or CHF 5633.

	*CHF 5633 (n = 7)*	*Poractant alfa (n = 8)*
Birth weight (kg)	2.71±0.14	2.78±0.14
Lung weight (g/kg)	33.34±1.50	32.00±2.70
Umbilical artery pH	7.38±0.03	7.29±0.03
*Differential cell counts in BALF (x 10^6^/kg bw):*		
Lymphocytes	2.55±0.48	1.54±0.19
Neutrophils	25.90±8.09^*^	72.81±11.22
Monocytes	21.97±6.95	30.85±4.35
**BALF hemoglobin (OD at 410 nm/kg bw)**	170.7±36.32	201.0±54.46

Neutrophils were decreased in the lungs of preterm lambs treated with CHF 5633 compared to Poractant alfa treated lambs after surfactant inactivation by albumin. BALF – bronchoalveolar lavage fluid; bw – bodyweight; OD – optical density. Data expressed as mean±SEM. * p<0.05, unpaired t-test.

**Figure 2:**
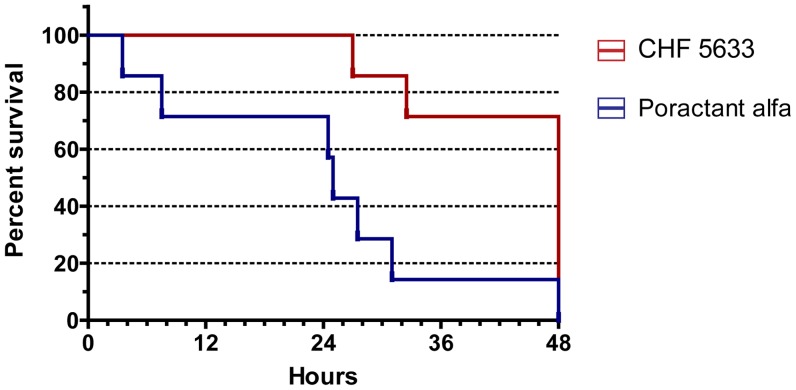
Survival. Kaplan-Meier curves represent the 48 hour survival of preterm lambs which underwent surfactant inactivation when treated either with CHF 5633 (dotted line) or Poractant alfa (continuous line). Analysis by Gehan-Breslow-Wilcoxon test indicated a significant longer survival of lambs treated with CHF 5633 compared to Poractant alfa treated lambs (p<0.05).

Lambs that were treated with CHF 5633 were more likely to survive up to 48 hours after birth (46.5 hours after albumin instillation) than lambs treated with Poractant alfa (5 out of 7 lambs treated with CHF 5633, compared to 1 out of 8 Poractant alfa treated lambs) (Figure 2). The total amount of administered surfactant and frequency of redosing did not differ significantly between treatment groups. However, when the frequency of re-dosing was corrected for post-inactivation survival time (in hours), to correct for the poor survival of Poractant alfa treated lambs, surfactant requirement was significantly lower among the CHF 5633 treated lambs (Table 2). Minute volumes and tidal volumes were not statistically different between groups (Figure S1).

**Table 2:** Surfactant administration.

	*CHF 5633 (n = 7)*	*Poractant alfa (n = 8)*
Total surfactant (mg/kg bw)	900.00±222.50	1400.00±233.00
Frequency of surfactant redosing	3.00±1.11	5.50±1.17
**Frequency of surfactant re dosing corrected for hours of survival after inactivation**	0.08±0.03*	0.30±0.05

Bw – bodyweight. Data expressed as mean±SEM. * p<0.05, unpaired t-test.

Differential cell counts of the bronchoalveolar lavage fluid (BALF) indicated that there were significantly fewer neutrophils present in the alveolar spaces of the lungs of CHF 5633 treated lambs (Table 1). The amount of hemoglobin in the BALF did not differ between the two treatment groups.

**Figure 3:**
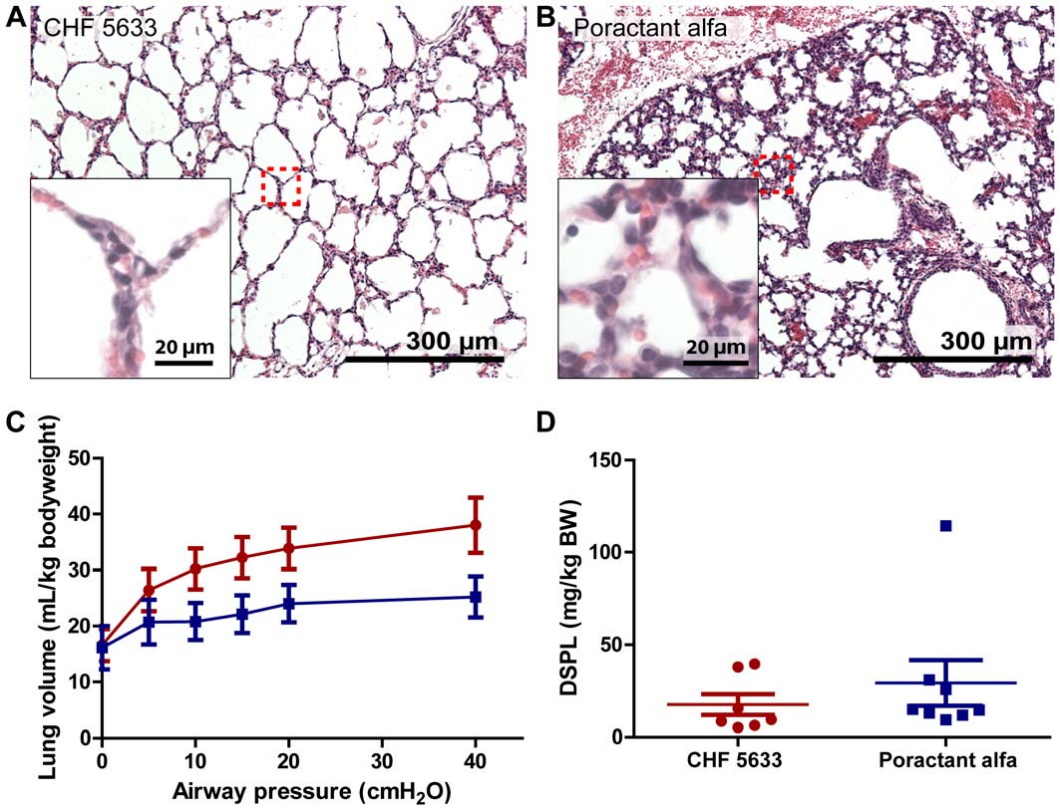
Lung physiology. Lung tissue stained with hematoxylin and eosin of lambs treated with CHF 5633 (**A**) and Poractant alfa (**B**). Alveolar spaces were much larger with less interstitial bleeding in lungs of lambs treated with the synthetic surfactant compared to the lungs of Poractant alfa treated lambs. **C** Lung compliance at 40 cmH_2_O airway pressure was increased in lambs treated with synthetic surfactant, but did not reach statistical significance (p = 0.056, two-way repeated-measures analysis of variance). **D** Disaturated phospholipids (DSPL) were not significantly different in the bronchoalveolar lavage fluid (BALF) of lambs treated with synthetic surfactant compared to the BALF of Poractant alfa treated lambs. Burgundy red spheres = CHF 5633; Blue cubes = Poractant alfa. Data expressed as mean±SEM. * p<0.05, Mann-Whitney u-test.

**Figure 4:**
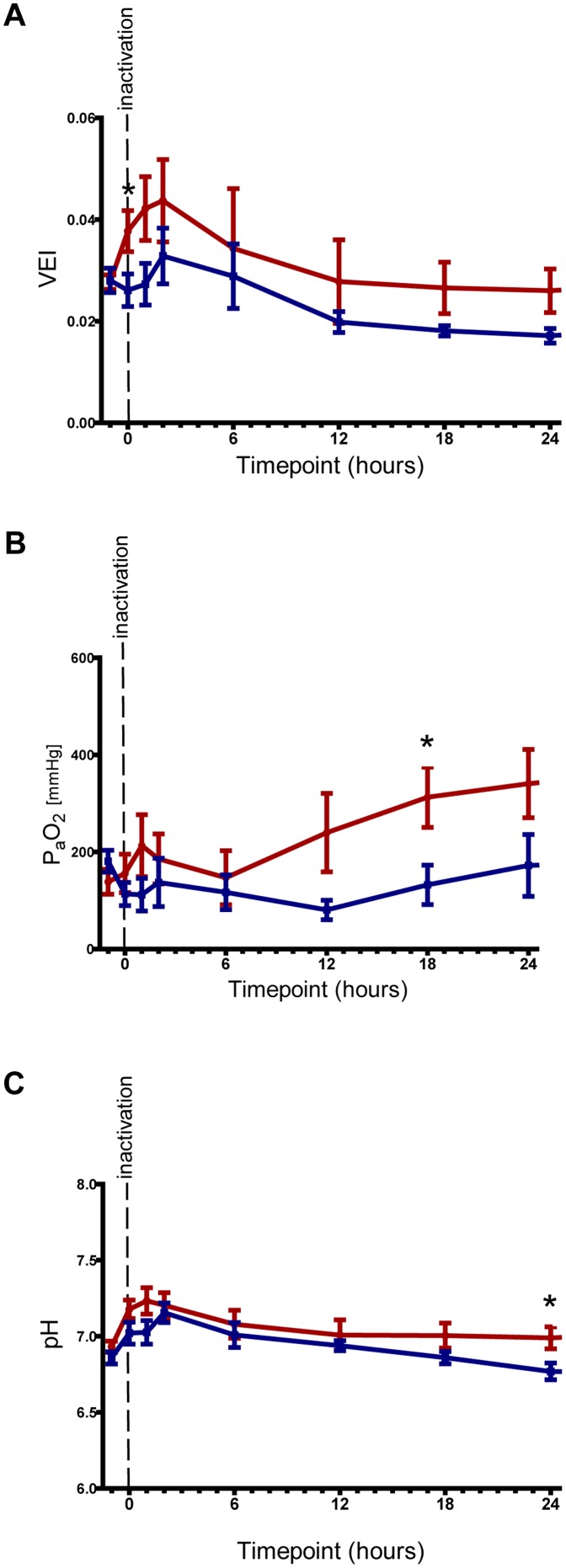
Ventilation. **A** The ventilation efficiency index (VEI) of lambs treated with CHF 5633 was elevated at all time points compared to Poractant alfa treated lambs, but only reached significance at 90 minutes after birth, just before inactivation (t = 0). **B** The partial oxygen pressure in arterial blood (P_a_O_2_) of lambs treated with CHF 5633 was elevated at most time points compared to Poractant alfa treated lambs, but only reached significance at 18 hours after surfactant inactivation. **C** The arterial blood pH of lambs treated with synthetic surfactant was elevated at all time points compared to Poractant alfa treated lambs, but only reached significance at 24 hours after surfactant inactivation. Grey spheres = CHF 5633; Black cubes = Poractant alfa. Data expressed as mean±SEM. *p<0.05, two-way repeated-measures analysis of variance.

Morphologically there was a striking difference between the lung structures of lambs treated with Poractant alfa or CHF 5633 (Figure 3A and 3B). The lung structure of lambs treated with CHF 5633 was more open, with larger alveolar spaces and thinner septa, whereas the lungs of Poractant alfa treated lambs did not inflate well, had thicker alveolar walls and contained more erythrocytes and lymphocytes. This difference in structure was reflected in the pressure volume curves (Figure 3C). The lungs of CHF 5633 treated lambs distended to a larger volume under increasing airway pressure compared to the lungs of Poractant alfa treated lambs, although this observation did not reach statistical significance. Disaturated phospholipids content in the BALF, which is an indicator for total surfactant content of the lung (both inherent and administered) did not differ between the two treatment groups (Figure 3D). However, these results may be confounded by the difference in survival rate of CHF 5633 treated lambs compared to Poractant alpha treated lambs, or differences in the amount of surfactant administered. In addition to these physiological parameters, the efficiency of surfactant treatment was determined by functional *in vivo* readouts. The ventilation efficiency index (VEI) and partial arterial oxygen pressure (P_a_O_2_), which could be calculated by combining ventilation parameters and blood gas measurements, revealed an increased VEI and P_a_O_2_ for animals treated with CHF 5633 during all time points (Figure 4A and 4B). Statistical significance was however only reached at 90 minutes of life for VEI and 16.5 hours after surfactant inactivation for P_a_O_2_. The pH of arterial blood showed a similar trend, and only reached statistical significance at 22.5 hours after surfactant inactivation. Because only one Poractant alfa treated lamb survived longer than 24 hours, statistical analysis of VEI, P_a_O_2_ and arterial pH was not possible for these parameters at 34.5 or 46.5 hours after surfactant inactivation.

**Figure 5:**
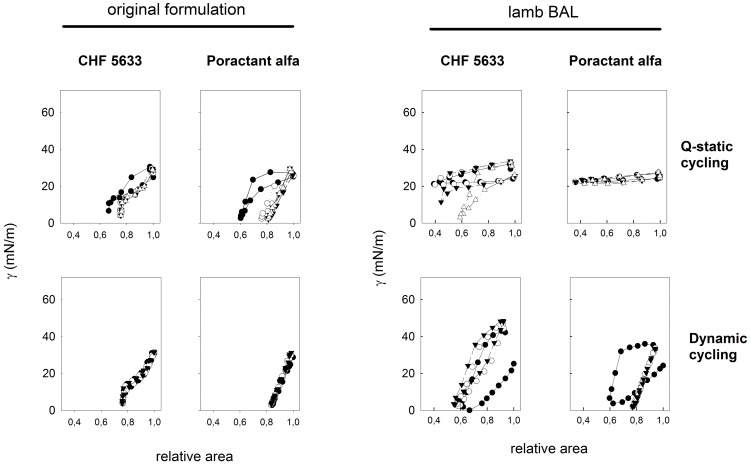
Surface behavior. Compression-expansion cycling isotherms in the CBS of films formed by original CHF 5633 or Poractant alfa preparations (left panels) and of films formed by material obtained from the bronchoalveolar lavage of CHF 5633-treated or Poractant alfa-treated animal lungs (right panels). Isotherms were obtained either under slow quasi-static (upper panels) or fast dynamic (lower panels) cycling regimes. Quasi-static isotherms include those from the 1^st^ (closed circles), 2^nd^ (open circles), 3^rd^ (closed triangles) and 4^th^ (open triangles) compression-expansion cycles, while dynamic isotherms are plotted from the 1^st^ (closed circles), 10^th^ (open circles) and 20^th^ (triangles) cycle.

To further detail the biophysical capabilities of surfactant from CHF 5633 treated or Poractant alpha treated animals, we compared the performance of surfactant complexes obtained from BALF of the different animals in the CBS. This technique has been widely used to evaluate the surface behavior of pulmonary surfactant films under conditions mimicking interfacial breathing mechanics [15], [24]. We compared illustrative compression-expansion isotherms of original CHF 5633 or Poractant alfa films and of films formed by surfactant pelleted from BALF of CHF 5633 treated or Poractant treated lungs (Figure 5). Both surfactant preparations were originally capable to form films able to reach very low tensions (below 5 mN/m) when subjected to compression-expansion cycling, either under quasi-static (slow) or dynamic (rapid, physiological-like) cycling regimes. Material from BALF of the surfactant treated animals was impaired with respect to their original capabilities. Surfactant from CHF 5633 treated animals was in most cases still able to produce very low tensions when films were subjected to either slow or fast compression-expansion cycling, although these films required slightly larger area compression than the original material ones (around 40% instead of 30%) to produce the minimal tensions. In contrast, films formed by lavage of Poractant-alfa treated lungs did not produce in most cases tensions below 20 mN/m when compressed at slow speed (Figure 5). However, when compressed at fast, physiologically-comparable rates, films from Poractant alfa treated animals underwent reorganization during the first compression cycle to produce films that were then able to produce minimal tensions with little compression in the subsequent cycles.

**Figure 6:**
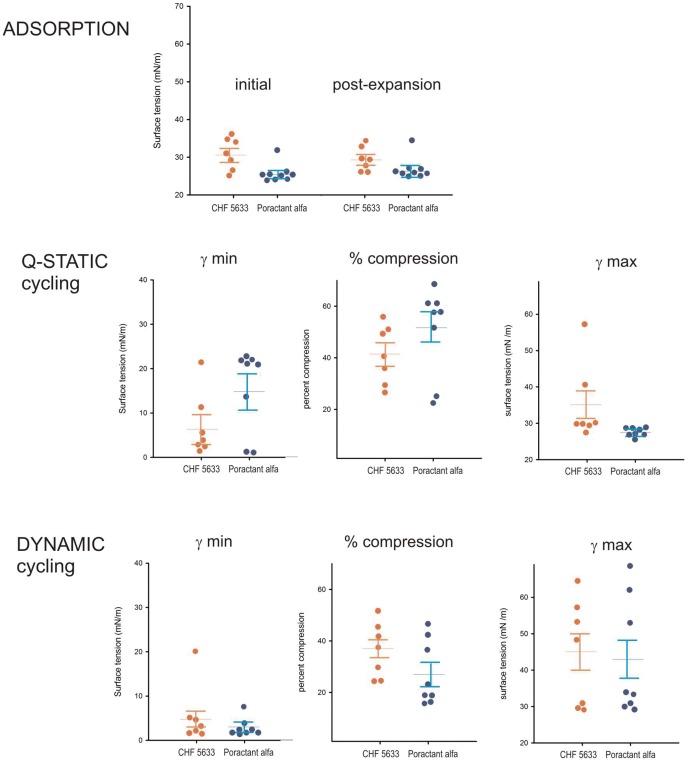
Parameters defining interfacial performance. Comparison of parameters defining the interfacial performance in the CBS of films formed by material obtained from the bronchoalveolar lavage of CHF 5633 treated and Poractant alfa treated animals. Data plotted are the minimal surface tension reached after 1 min of initial or post-expansion adsorption (upper panel), and the minimal surface tension, the percent of area reduction required to reach minimal tension and the maximal tension during quasi-static (middle panels) or dynamic (lower panels) compression-expansion cycling. Data expressed as mean±SEM. *p<0.05, two-way repeated-measures analysis of variance.

Relevant parameters obtained from compression-expansion isotherms of films formed by the different samples in the CBS were summarized in Figure 6. Surfactant from both CHF 5633 treated and Poractant-alfa treated animals adsorbed well to the air-water interface, producing equilibrium surface tensions ≤30 mN/m in less than a minute. Surfactant from Poractant alfa treated animals exhibited slightly faster adsorption than surfactant from CHF 5633 animals, a difference that was statistically significant. The larger difference in the surface performance of the two groups was observed upon cycling of films at slow speed. Slow quasi-static compression-expansion cycling usually reveals intrinsic differences in the compressibility properties of films made of different materials or having different organization [14], [24]. Films from CHF 5633 treated animals produced under quasi-static cycling in most cases much lower surface tensions, with less area reduction, compared to films of surfactant from Poractant alfa treated lungs. Differences in surface behavior practically vanished when the films were subjected to fast cycling at rates comparable to those potentially occurring in the lung in vivo.

## Discussion

Surfactant replacement therapy has been the most significant advance in perinatal care to decrease neonatal mortality since the late 1980's, equaled only by antenatal corticosteroids [26]. The chances of survival after preterm birth and low morbidity increased dramatically in the past decades. Surfactant replacement therapy is established and well-studied. However, the inherent costs of surfactant preparations and the risk of inactivation urge the search for new surfactant preparations. Been et al. for example found that the exposure to antenatal inflammation (chorioamnionitis) resulted in a poor response to exogenous surfactant replacement therapy [27]. This is a clinical relevant example that the success of surfactant replacement therapy can be reduced by inactivation due to inflammatory changes in the lung. The dose of surfactant and/or the preparation of the surfactant might present clinical alternatives in the best clinical care after preterm birth.

We developed a model to study surfactant inactivation in premature, virtually surfactant deficient lambs and showed that CHF 5633 has a survival benefit and a trend for improved ventilation and oxygenation after *in vivo* inactivation. An additional benefit of CHF 5633 is that it had a more durable effect, as re-dosing was not required as frequently as in the Poractant alfa treated lambs. To our information this is the first study that shows superiority of a surfactant preparation for inactivation *in vivo*. Albumin was chosen as inhibitor since it is well studied as inducer of surfactant inactivation *in vitro* and *in vivo* [28], [29]. In addition, albumin is a plasma protein and part of the inflammatory cascade induced by various stimuli such as mechanical ventilation or infection [30], [31]. The inhibitory effect of albumin was clearly seen during our experiments as most lambs, particularly the ones treated with the conventional Poractant alfa surfactant, showed a decrease in arterial oxygenation levels and ventilation efficiency index shortly after albumin instillation.

The study has several limitations. First of all, the sudden onset of inactivation after birth (besides the injury caused by mechanical ventilation) is not very common in clinical practice as are the ventilation strategies used in this model. However, we chose the setup of sudden inactivation and aggressive ventilation strategies to reduce variability between individual animals and study the effects of the two surfactant preparations in the most unfavorable conditions. Secondly, we have limited the follow-up to 48 hours, which is rather long for an experimental study but still far away from clinical practice. Furthermore, we did not include control animals treated with CHF 5633 or Poractant alfa only nor test different doses of CHF 5633 or inhibitors other than albumin.

CHF 5633 was developed with properly designed synthetic analogs of the two natural SP-B and SP-C proteins. Natural surfactant as produced by the lungs consists of a complex mixture of several phospholipids and the proteins SP-A, SP-B, SP-C and SP-D, of which SP-B and SP-C play a key role in decreasing surface tension in the lung [14], [32]. Experimental research showed that the inclusion of both SP-B and SP-C in exogenous surfactant preparations improved the biophysical effects in preterm rabbits [33]. The addition of SP-B to natural surfactant preparations that already contained SP-B further improved the function *in vivo* [34]. The experience with the importance of both SP-B and SP-C in natural surfactants has been repeated with synthetic surfactants. Most previously reported synthetic surfactants either contain SP-B or SP-C [35]–[37], but for an optimal functional effect both proteins are required. Almlén and colleagues [38], [39], have demonstrated that a synthetic preparation containing SP-B and SP-C was superior to single-peptide surfactants in premature ventilated rabbits. Our study can now confirm that a similar synthetic preparation has proven to have superior resistance to inactivation as well.

Our biophysical measurements suggest that the higher resistance of CHF 5633 surfactant to inactivation *in vivo* could be related to better compressibility properties of CHF 5633 films after exposure to inflamed and injured lungs. *In vitro* studies established that the main effect of albumin as a surfactant-inactivating agent could be related to impairment of the ability of surfactant to form surface active films at the air-liquid interface, as a consequence of the competition of albumin and surfactant to reach the interface. However, the impairment of surface activity observed in the surfactant from the BALF from our lambs, particularly in the case of Poractant alfa, resembles the impairment of surfactant due to incorporation of inhibitory substances such as cholesterol or bile acids [13]. Interfacial adsorption was not impaired in surfactant from Poractant alfa treated animals, but was even slightly better than that from CHF 5633 treated lambs, probably because the fluidization of Poractant alfa that impairs compressibility and favors adsorption. This suggests that the introduction of albumin into the lungs causes surfactant inactivation not so much due to the primary impairment of interfacial adsorption but also indirectly through the release of some surfactant inhibitors as a consequence of lung inflammation. In these conditions surfactant films become highly deformable and are not able to reach the lowest tensions (very high surface pressure) when subjected to compression. Our data suggest that either Poractant alfa is more susceptible than CHF 5633 to incorporate spurious components leaked to the airspaces, or that the particular lipid and protein composition of CHF 5633 can accept higher amounts of inhibitors before losing the compressibility properties required to reach and sustain the low surface tensions required to stabilize the lungs. Further studies are required to understand the origin of the different inhibitory susceptibility, but the apparently higher resistance to inhibition of CHF 5633 may provide improved treatment opportunities not only for preterm infants who have decreased oxygenation due to surfactant inactivation by inflammation, but also for patients suffering of Acute Respiratory Distress Syndrome (ARDS).

In conclusion, this is the first study which shows that a synthetic surfactant with properly designed analogs of SP-B and SP-C results in a similar oxygenation in preterm lambs but also conveys a survival benefit to preterm lambs over the gold standard treatment Poractant alfa with the same doses. The promising results reported here support the introduction of CHF 5633 as a possible new therapy for surfactant deficiency or dysfunction conditions. The superiority in this model will have to be confirmed in a clinical trial.

## Supporting Information

Figure S1 **Minute and tidal volume.** Recordings of the minute (**A**) and tidal volume (**B**) did not show any significantly difference between CHF 5633 treated animals and Poractant alfa treated animals for the duration of the experiment. Grey spheres = CHF 5633; Black cubes = Poractant alfa. Data expressed as mean±SEM. *p<0.05, two-way repeated-measures analysis of variance.
(TIF)
