# Supplementary figures and images for: New Surfactant with SP-B and C Analogs Gives Survival Benefit after Inactivation in Preterm Lambs
Source: PLoS One. 2012 Oct 16;7(10):e47631. doi: 10.1371/journal.pone.0047631 (PMC3473048; doi:10.1371/journal.pone.0047631)

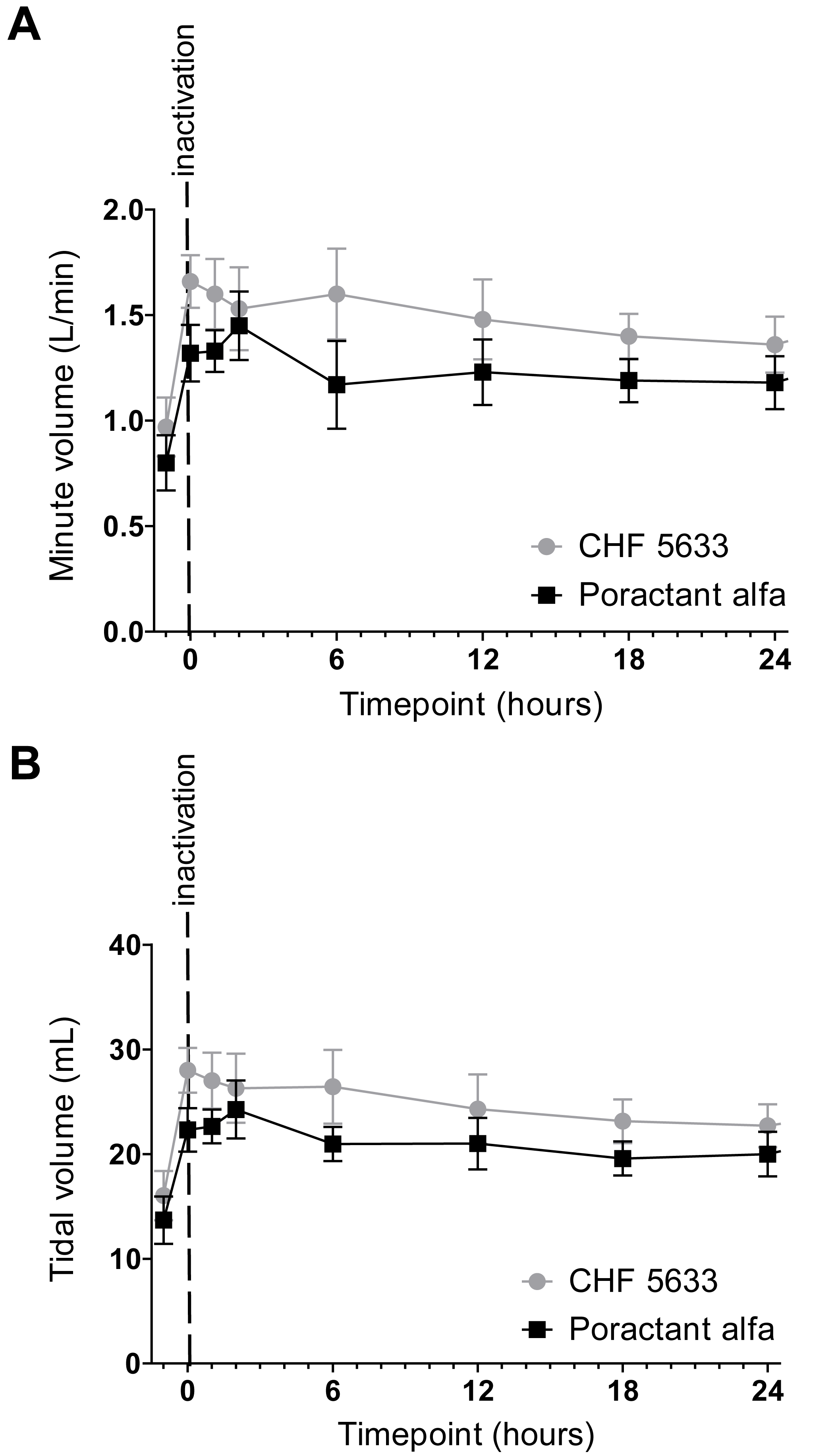

Supplement: Figure S1 — Minute and tidal volume. Recordings of the minute (A) and tidal volume (B) did not show any significantly difference between CHF 5633 treated animals and Poractant alfa treated animals for the duration of the experiment. Grey spheres = CHF 5633; Black cubes = Poractant alfa. Data expressed as mean±SEM. *p<0.05, two-way repeated-measures analysis of variance. (TIF) [file pone.0047631.s001.tif]
